# Neural Substrates of Transcutaneous Spinal Cord Stimulation: Neuromodulation across Multiple Segments of the Spinal Cord

**DOI:** 10.3390/jcm11030639

**Published:** 2022-01-27

**Authors:** Trevor S. Barss, Behdad Parhizi, Jane Porter, Vivian K. Mushahwar

**Affiliations:** 1 Neuroscience and Mental Health Institute, University of Alberta, Edmonton, AB T6G 2R3, Canada; tbarss@ualberta.ca (T.S.B.); parhizi@ualberta.ca (B.P.); 2 Division of Physical Medicine and Rehabilitation, Department of Medicine, University of Alberta, Edmonton, AB T6G 2R3, Canada; jporter@ualberta.ca; 3 Sensory Motor Adaptive Rehabilitation Technology (SMART) Network, University of Alberta, Edmonton, AB T6G 2R3, Canada

**Keywords:** neuromodulation, interlimb coordination, rehabilitation, neurophysiology, Hoffmann (H)-reflex, motor-evoked potential, locomotion, spinal cord injury

## Abstract

Transcutaneous spinal cord stimulation (tSCS) has the potential to promote improved sensorimotor rehabilitation by modulating the circuitry of the spinal cord non-invasively. Little is currently known about how cervical or lumbar tSCS influences the excitability of spinal and corticospinal networks, or whether the synergistic effects of multi-segmental tSCS occur between remote segments of the spinal cord. The aim of this review is to describe the emergence and development of tSCS as a novel method to modulate the spinal cord, while highlighting the effectiveness of tSCS in improving sensorimotor recovery after spinal cord injury. This review underscores the ability of single-site tSCS to alter excitability across multiple segments of the spinal cord, while multiple sites of tSCS converge to facilitate spinal reflex and corticospinal networks. Finally, the potential and current limitations for engaging cervical and lumbar spinal cord networks through tSCS to enhance the effectiveness of rehabilitation interventions are discussed. Further mechanistic work is needed in order to optimize targeted rehabilitation strategies and improve clinical outcomes.

## 1. Introduction

Neuromodulation of the spinal cord by means of non-invasive transcutaneous (tSCS) and implanted epidural (eSCS) spinal cord stimulation may improve sensorimotor rehabilitation after spinal cord injury (SCI) [1,2,3,4]. However, developing an optimal treatment approach requires taking advantage of the intrinsic ability of the spinal circuits by facilitating preserved sensorimotor pathways that could drive spinal plasticity [5]. The influence of spinal cord stimulation (SCS) does not necessarily depend on the nature of the neurological disorder, but on the operational and functional status of residual neural networks [6]. Epidural SCS has been shown to modulate neuronal circuits in persons with motor-complete SCI, including corticospinal, [7,8,9] propriospinal [10,11], and corticoreticulospinal [12] tracts. The resulting neuroplasticity is thought to improve spinal motor output and volitional movements even in cases of severely reduced supraspinal input, without negatively impacting residual motor function [3,13,14,15,16,17]. Most recently, eSCS applied to the lumbar spinal cord, in conjunction with intensive locomotor training, enabled persons with clinically motor-complete SCI to walk over ground for short distances [4,13,18]. This demonstrates that dormant neurons that survive the injury may be reengaged with spinal neuromodulation, and can produce stepping-like movements [19,20].

While eSCS has important implications for rehabilitation after SCI, its invasive nature, high cost, and limited accessibility are limitations for rapid translation to a broad population. Transcutaneous SCS is a non-invasive, accessible, and cost-effective alternative that is thought to be a safe assistive technology with important implications for both furthering our understanding of the mechanisms controlling locomotion, and for rehabilitating sensorimotor function after SCI [21,22,23]. It has been suggested that tSCS of the lumbar spinal cord may activate similar spinal circuitry to eSCS [24,25,26]; if accurate, tSCS is likely to enhance functional recovery in a similar manner to eSCS when paired with rehabilitation strategies. This would also allow for the tSCS to build on the foundation of knowledge of the intrinsic circuitry recruited by eSCS. In case studies and small clinical trials, tSCS improved hand and arm function [2,27,28,29], produced locomotor-like stepping [1,30], and improved walking function [22,31,32] in participants with neurological deficits including incomplete and complete SCI, stroke, and cerebral palsy. Evidence suggests that tSCS may also be used as a viable alternative to pharmacological anti-spasticity approaches, altering the excitability of spinal pathways and possibly augmenting pre- and post-synaptic inhibitory mechanisms [33,34]. Understanding the impact that tSCS has on spinal cord circuitry is vital to ensuring that the stimulation is applied at therapeutically appropriate sites, and that the parameters of stimulation are chosen so as to optimize the desired rehabilitative effects.

It is critical to realize that not all of the studies using tSCS follow the same pattern of stimulation. Transcutaneous SCS patterns including single pulses, trains of pulses, and waveforms with and without carrier frequencies have been used. The present review focuses on the use of alternating current (AC) tSCS, because most studies aimed at improving functional recovery after SCI have used this type of stimulation. Direct current (DC) tSCS also modulates spinal excitability, and may be another promising and novel tool to pair with activity-based interventions [35,36]; however, this technique is beyond the scope of this review, and requires further research in order to determine the specific mechanisms involved. In this review, the two common patterns of AC tSCS that have been employed to date will be included and discussed in detail. The first pattern, which will be referred to as unmodulated tSCS, does not include a carrier frequency, and is generally composed of rectangular pulses delivered as single individual pulses, or in trains of 1–90 Hz frequency. The second stimulation pattern, which will be referred to as modulated tSCS, includes rectangular pulses with a carrier frequency of 2.5–10 kHz, delivered at a rate of 5–40 Hz [37]. While both patterns have been reported to modulate neural circuitry across the central nervous system and produce functional outcomes, it is unlikely that they share identical mechanisms of action. The fundamental differences between the two patterns will be highlighted throughout this review as the different studies are discussed.

The aims of this review are as follows: first, to identify the parameters and the potential underlying mechanisms that allow tSCS to facilitate ongoing motor output; secondly, to highlight the effects of tSCS on excitability across multiple segments of the spinal cord; thirdly, to address the ability of multiple sites of tSCS to converge and enhance modulation of spinal reflex and corticospinal pathways; and finally, to explore the potential and limitations for engaging cervical and lumbar spinal cord networks through tSCS to enhance the effectiveness of rehabilitation interventions. This review will also underscore the need for further mechanistic work to optimize tSCS parameters that, when paired with targeted rehabilitation strategies, can effectively improve clinical outcomes.

## 2. Historical Perspective

The use of electricity for neuromodulation has a storied history, ultimately leading to a variety of therapeutic electrical stimulation techniques that target spinal networks, including tSCS, eSCS, and intraspinal microstimulation (ISMS) [38,39]. Epidural SCS initially emerged in the pain literature in 1967 [40], and is currently most commonly used for the treatment of intractable chronic pain; while originally designed to alleviate pain, it was used in 1971 as a method for facilitating motor control in persons with multiple sclerosis [41], and to reduce spasticity after incomplete SCI [42,43,44]. In 1979, tonic stimulation of dorsal roots of the spinal cord was shown to generate locomotion in low-spinal cats [45,46]. This work then led to initial investigations demonstrating improved stepping in humans, and providing the potential for this technology to be used as a translational tool to facilitate improved function after neural injury [3,15,47,48].

In humans, eSCS involves implanting electrodes over the dura mater encasing the lumbosacral segments of the spinal cord. Dorsal root fibers are the first to be recruited, with the lowest thresholds, while the ventral root fibers are the least accessible [49]. This recruitment leads to the activation of motor neurons through monosynaptic and polysynaptic proprioceptive circuits, and increases the overall excitability of the spinal cord, allowing for greater responsiveness of spinal circuits to descending signals and sensory feedback [14]. Extensive evidence from animal studies has led to the hypothesis that electrically stimulating the human spinal cord through the epidural space can facilitate improvements in motor function.

Transcutaneous SCS was inspired by high-voltage percutaneous electrical stimulation over the lumbosacral spinal column to activate peripheral motor axons [50]. In 1997, the generation of locomotor-like activity with the application of tSCS over the lumbar enlargement was demonstrated in individuals with SCI [51]. It was then suggested that there are low-threshold sites in the posterior structure of the human lumbosacral cord that could be accessed from the surface [49]. In 2007, and encouraged by earlier discoveries, Minassian et al. revealed that posterior root afferents can be accessed by tSCS with single pulses (unmodulated), and they reported monosynaptic reflex responses in multiple muscles of the legs [52]. Later, it was shown that unmodulated tSCS can enhance voluntary locomotor-like electromyographic (EMG) activity [53] and modify spasticity in individuals with incomplete SCI [54]. In 2015, tSCS was used with a novel waveform that included a carrier frequency (i.e., modulated) to activate spinal networks while reducing the perception of pain associated with the necessarily high stimulus amplitudes [22,30]. The tSCS parameters were based on a previous finding that a 10 kHz carrier frequency of transcutaneous stimulation reduces the likelihood of activating pain fibers [55]. Building on these exciting initial investigations, the tSCS literature has incorporated a diverse set of stimulation parameters that are vital to understand, as they may have important implications for improving function in persons experiencing sensorimotor impairments due to neurological conditions.

## 3. Properties of Transcutaneous Spinal Cord Stimulation (tSCS)

### 3.1. Parameters of tSCS

**Figure 1:**
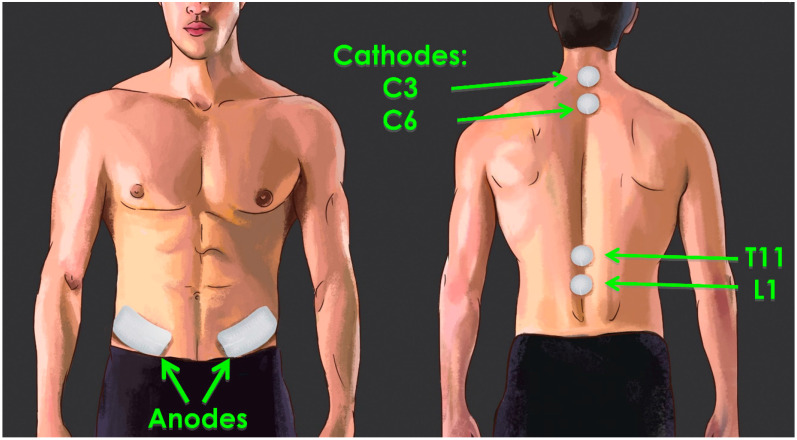
Typical tSCS electrode placement: Transcutaneous SCS is commonly delivered via two 2.5 cm round cathodic electrodes placed over the C3–4 and C6–7 (cervical) or T11 and L1 (lumbar) spinous processes. Two 5 × 10 cm rectangular anodic electrodes are placed bilaterally over the iliac crests.

Typically, tSCS is applied through circular adhesive electrodes of 2–3 cm diameter that are placed on the skin overlying the lumbar or cervical segments of the spinal cord (Figure 1). Optimal placement of electrodes is dependent on the individual symptoms, desired rehabilitation outcomes, and paired rehabilitation strategies, on a case-by-case basis. When targeting the lower extremities, the most common cathode placement is over the T11–T12 and/or L1–L2 spinous processes, while C6–C7 or C7–T1 is the most common placement for the upper extremities [37]. The anode electrodes are placed either over the iliac crests or the anterior superior iliac spine [37].

In addition to electrode placement, it is important to consider the waveform characteristics of the applied current for maximal therapeutic outcomes [5,17,21]. With unmodulated tSCS, which evolved from the eSCS literature, rectangular mono- or biphasic pulses of 0.4–2 ms duration are typically delivered at a frequency range of 1–90 Hz and stimulation intensity of up to 170 mA [21,37]. On the other hand, the novelty of the modulated stimulation pattern comes from its unique waveform, which includes a carrier frequency of up to 10 kHz within a given pulse. Such high-frequency stimulation approaches were originally used to reduce the perception of pain during transcutaneous nerve stimulation [55]. The waveform in the modulated stimulation pattern generally consists of 0.3–1 ms long rectangular biphasic or monophasic pulses that repeat at a frequency of 5–40 Hz. Each of these pulses encompasses a carrier frequency of 2.5–10 kHz, aimed at suppressing the user’s perceived pain and, thus, allowing for greater current amplitudes to be employed. The amplitude of the current for modulated tSCS is similar to that of unmodulated tSCS, and ranges from 30 to 180 mA, depending on the stimulation site and the desired outcome. In neurologically intact participants, the intensity of modulated tSCS (with a 5 kHz carrier frequency) allows for maximal tolerable current amplitudes of 103 mA, while unmodulated tSCS has maximal tolerable amplitudes of 39 mA. However, when considering maximal tolerable stimulation with respect to the stimulation levels needed to evoke motor responses, tSCS with a carrier frequency was no different than unmodulated tSCS in reducing the perception of pain [56].

Interestingly, when using an array of electrodes and adjusting the parameters of stimulation—including intensity and location—different patterns of independent and coordinated upper limb motion at both distal and proximal joints have been elicited, showing the potential of tSCS without a carrier frequency to evoke functional movements [28]. Therefore, the chosen parameters of tSCS can have a meaningful effect on the recruited circuitry and the functional movements that are facilitated or inhibited. Understanding how the applied electrical current is integrated into the spinal circuitry is vital.

### 3.2. Current Flow Involved in tSCS

**Figure 2:**
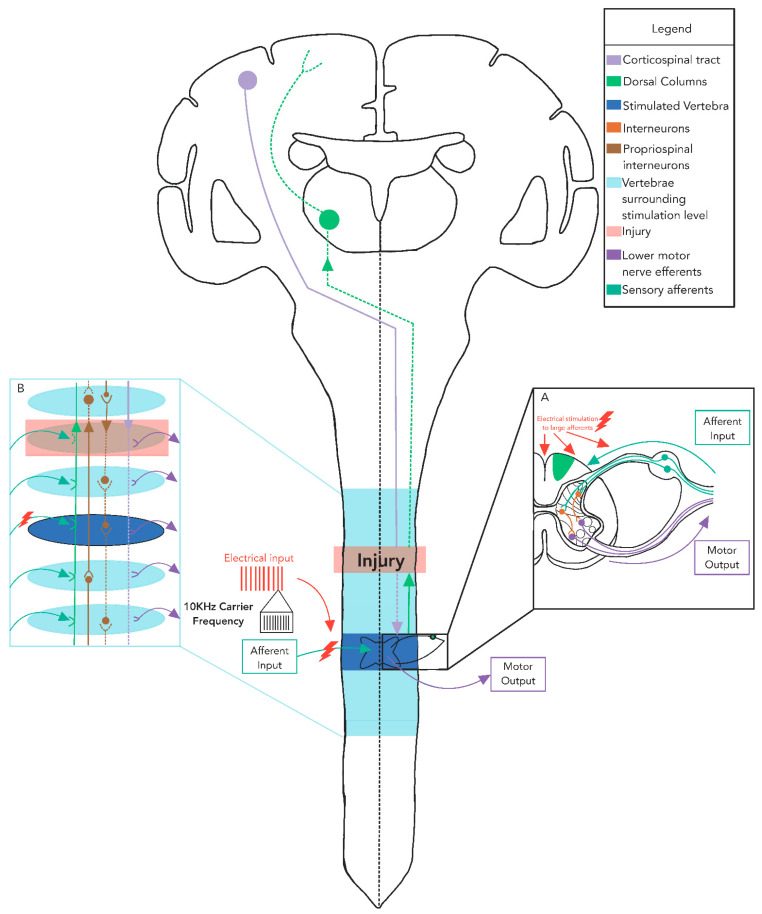
Schematic of networks within the spinal cord that are potentially altered with tSCS: The main figure highlights the ability of tSCS to modulate ongoing motor output through dorsal root afferents that trans-synaptically facilitate motor output by bringing previously inaccessible motor units closer to their threshold, allowing them to contribute to the execution of a desired task. (**A**) Large-diameter afferents are likely activated and synapse on several types of interneurons that facilitate ongoing motor output. (**B**) Among these interneurons are propriospinal interneurons, which transmit this input to multiple segments of the spinal cord in order to alter excitability and impact ongoing motor output throughout the cord. Solid lines indicate that transmission remains intact to the point of injury to the spinal cord, while dashed lines indicate that transmission is impaired, and may be facilitated by tSCS. Typically, tSCS is applied in single unmodulated or modulated monophasic or biphasic pulses or trains of pulses.

The current flow and electrical potential generated by eSCS and tSCS are markedly different [24,25]. With eSCS, 80–90% of the ionic current flows between the active electrodes through the cerebrospinal fluid [57]. In tSCS, the current flow is strongly influenced by the electrical properties of the numerous conductivity boundaries of body tissues (e.g., skin, fat, muscle, and bone), with computer simulations estimating that only ~8% of the overall current flows through the cerebrospinal fluid [25]. With the dramatic difference in current flow and the proximity of neural structures to the electrodes between eSCS and tSCS, both the selectivity of spinal circuitry and the required stimulation intensity are dissimilar. Modelling studies suggest that the superficially located large-diameter posterior column fibers with multiple collaterals have a threshold three times higher than that of posterior root fibers [26]. For both tSCS and eSCS, large-diameter proprioceptive sensory fibers within the posterior rootlets/roots have the lowest thresholds of all neural structures within the vertebral canal [24], making it unlikely that the effects of SCS arise exclusively from dorsal column stimulation [57]. Computer modeling indicates that action potentials generated by tSCS are initiated in the posterior root fibers at their entry into the spinal cord, or along the longitudinal portions of the afferent fiber trajectories, depending on the cathode position [25]. Evidence suggests that the reflex nature of unmodulated tSCS exploits the difference in the strength–duration properties of sensory and motor axons; however, future research should be conducted to explore how modulated tSCS generates action potentials in neural tissue [58]. At stimulation intensities that result in the recruitment of posterior column axons, co-activation of posterior root fibers of large and small diameters is observed, demonstrating the substantial differences in the thresholds of activation of various components of the spinal cord [26]. Moreover, increasing stimulation intensity engages spinal interneurons via synaptic projections which, in turn, activate motor neurons [22,59]. These simulation results provide a biophysical explanation for the electrophysiological findings of lower limb muscle responses that are induced by posterior root stimulation (Figure 2A). However, it should be noted that these computer simulation studies have all applied unmodulated tSCS (i.e., without carrier frequency), and the results may not necessarily be generalizable to other types of pulses. Understanding the potential unique properties associated with modulated tSCS is vital for implementing tSCS in a manner that optimizes functional recovery after neural injury or disease. Similar simulation studies using high carrier frequencies are necessary in order to extend the knowledge regarding current flow in tSCS.

### 3.3. Transcutaneous SCS Carrier Frequency Is Important for Reducing Discomfort, but Its Role in Restoring Motor Function Remains Unclear

The inclusion of a carrier frequency within a given stimulation pulse is used for its ability to disrupt synchronous firing of the high-threshold C-fibers related to pain perception [60]. Pain management through SCS is based on the gate control theory introduced in 1965 [57], which proposed that the activation of Aβ mechanoreceptor fibers that synapse onto a range of neurons within the dorsal horn that release inhibitory neurotransmitters—including γ-amino butyric acid (GABA) and adenosine [61]—reduces the activity of nociceptive projection neurons in laminae I and V traveling along the spinothalamic tract. It has also been proposed that high-frequency stimulation of the spinal cord blocks discomfort by inactivating paresthesia-inducing large-diameter fibers and activating medium–small-diameter fibers that suppress wide-dynamic-range neurons encoding neuropathic pain [62]. Sub-perception SCS at 1 kHz was more effective for pain relief compared to low-frequency supra-perception stimulation [63]. Moreover, a recent eSCS study suggested that there was no observable difference between 1 kHz and 10 kHz stimulation for the relief of back pain [64]. Charge per pulse is lower in high-frequency eSCS in comparison with low-frequency stimulation, while charge per second is higher [61]. While these studies did not use tSCS, and were only aimed at pain management, they can play an important role in explaining the potential mechanisms that reduce discomfort in modulated tSCS. Manson et al. have recently shown that the maximal tolerable stimulation intensity is significantly greater during modulated tSCS compared to unmodulated tSCS [56]; however, the stimulation intensity required to evoke a muscle response (motor threshold) was correspondingly higher with a carrier frequency, leading to no difference in the relative current amplitude required to evoke a motor response [56]. This study indicated that the addition of a carrier frequency reduces discomfort for a given current amplitude compared to unmodulated tSCS, but does not reduce discomfort when evoking the same motor response.

What is less clear is the impact that the carrier frequency has on the neural circuitry recruited during tSCS, and the specific role it serves to improve functional recovery when paired with rehabilitation strategies. Recently, hand and arm function improved significantly during a single session of cervical tSCS with a 5 kHz carrier frequency applied in individuals with an SCI compared to when a carrier frequency was not included [65]. However, limited data are available as to the differences in specific neural substrates recruited by tSCS with and without a carrier frequency. Overall, integrating a carrier frequency may be an important feature of tSCS that not only circumvents pain compared to other stimulation profiles, but also promotes effective restoration of function after SCI. Further exploration is required in order to understand whether the carrier frequency is a unique feature necessary for optimizing the use of tSCS for sensorimotor recovery. Incorporating this knowledge into a mechanistic framework for the implementation of tSCS is essential in order to facilitate optimal functional recovery after neurological damage.

### 3.4. Mechanisms of tSCS Recruitment

The principal mechanism by which tSCS non-invasively activates inaccessible neuronal networks of the spinal cord likely includes the recruitment of afferent fibers (large–medium) in the posterior root in order to elevate spinal network excitability [66,67]. The excitability of spinal interneuronal networks can be readily modulated (changing the networks’ physiological state) without directly producing action potentials [22]. The route of stimulation propagation is through the dorsal root afferents, as indicated by the significant inhibition of cervical tSCS responses when using paired stimuli, during passive muscle stretching, and during muscle–tendon vibration [67]. Moreover, it has been suggested that eSCS and modulated tSCS can engage both afferent and efferent pathways, based on observations of early- and medium-response components of evoked potentials that are partially ascribed to posterior roots/group Ia/group II and motor neurons/anterior roots [22,68]. It is proposed that as stimulation intensity is increased, in addition to the Ia afferents, the smaller diameter afferents such as group Ib, larger diameter cutaneous afferents, group II muscle spindle afferents, and even more intraspinal connections and spinal interneurons are recruited through tSCS, similarly to what has been observed in eSCS [22,59]. This, in turn, brings interneurons and motor neurons closer to their firing threshold, making them more likely to respond to limited post-injury descending drive and improving supraspinal control after both modulated and unmodulated tSCS [24,30]. Both electrophysiological and computer modeling studies to date suggest that unmodulated tSCS excites posterior root fibers similarly to eSCS [24,52].

Recently, a few studies have compared the different effects of modulated and unmodulated tSCS on descending input. Benavides et al. reported that single-site tSCS applied with a 5 kHz carrier frequency at the C5–C6 level facilitated the amplitude of cervicomedullary-evoked potentials (CMEPs), but did not increase the amplitude of the motor-evoked potentials (MEPs) [65]; this was accompanied by an increase in the level of short-interval cortical inhibition (SICI). When tSCS was applied without the carrier frequency, both cortically and subcortically driven responses were facilitated. This is similar to our recent investigation, which found that modulated tSCS (33 Hz trains of 1 m long pulses with a 10 kHz carrier frequency) applied over the C3–4 and C6–7 spinous processes in neurologically intact individuals did not alter MEPs assessed in the forearm flexors [69]. Moreover, data from a paired associative stimulation (PAS) paradigm involving single pulses of transcranial magnetic stimulation (TMS) and unmodulated tSCS arriving at the same time at spinal motor neurons revealed increases in corticospinal excitability, but facilitation of MEPs following tSCS was less pronounced when tSCS pulses were filled with a carrier frequency [70]. These studies highlight the fact that in the presence of a carrier frequency, tSCS may be unable to facilitate MEPs. In contrast, it was shown that sub-motor-threshold tSCS without a carrier frequency, applied for a short period of 10 min to the cervical region, did not alter the excitability of the corticospinal and spinal reflex pathways [71]. At first glance, these results seem contradictory; however, the stimulation duration, stimulation amplitude, frequency of stimulation, stimulation waveform (modulated/unmodulated), and target muscles varied across these studies, which may have influenced the neuromodulatory effects of tSCS. By priming neural structures at the level of the spinal cord, unmodulated tSCS modulated spinal reflex excitability and reduced spasticity in a manner similar to that seen with passive cycling movements [34]. This suggests that alterations in spinal circuitry—including presynaptic influences—are likely the primary target of tSCS, and play an important role in the recovery of arm and hand function in persons with SCI.

Importantly, dorsal root stimulation is likely not entirely responsible for the effects of tSCS. Group Ia muscle spindle afferent fibres, which travel in the dorsal roots, have a lower threshold of activation compared to the largest cutaneous fibres [72]. If the effects of tSCS are only due to the activation of dorsal root afferents, then at low stimulation amplitudes the large-diameter group Ia afferents should be activated, leading to muscle contractions and proprioceptive errors via monosynaptic reflexes [57]. However, cutaneous sensation typically occurs over a large range of stimulus amplitudes that are lower than those required to produce motor responses mediated by purely monosynaptic reflex pathways, and proprioceptive errors are not a significant occurrence [73], making it unlikely that tSCS functions entirely by stimulating dorsal root afferents. Epidural SCS at 1–2 Hz has been shown to activate inhibitory interneurons in laminae I–III, albeit with latencies consistent with trans-synaptic (i.e., indirect) activation [74]. Therefore, it is important to consider whether inhibitory neurons in this region are the main or, at least, a contributing mechanism underlying the therapeutic benefit of tSCS; that is, tSCS may restore inhibition by enhancing dorsal horn GABAergic systems. It has been suggested that islet cells in the substantia gelatinosa require further consideration as prime candidates for the inhibitory effects on pain [57].

Moreover, while it is widely believed that tSCS depolarizes sensory afferents in the dorsal roots and dorsal horn that trans-synaptically recruit motor pools, it remains possible that polysynaptic connections from cutaneous mechanoreceptors in the skin act on both sensory processes and motor pools in the spinal cord. This, in turn, alters the excitability at both the level of the spinal cord—where the stimulation is provided—as well as remote levels of the spinal cord, through propriospinal interneuronal connections. Cutaneous inputs are known to have diffuse input that is specific to the task, phase, and amplitude at which stimulation is delivered [75,76]. It is therefore plausible that the recruitment of cutaneous mechanoreceptors surrounding the electrodes may contribute to the neuromodulatory effects of tSCS through these polysynaptic connections. The potential role of cutaneous mechanoreceptors in the skin with tSCS remains an important avenue to explore in future work [77,78,79].

A potential mechanism by which tSCS improves upon previously developed rehabilitation interventions is potentiation. Guiho et al. observed potentiation of supraspinal evoked responses with both dorsal eSCS and modulated tSCS over the C3–4 and C7–T1 intervertebral spaces in monkeys, but facilitation was stronger with dorsal eSCS [80]. It is vital to identify the capability of unmodulated tSCS to alter supraspinally driven responses compared to eSCS and modulated tSCS, in order to identify whether unique stimulation parameters are required for individual outcomes. Similarly, PAS with tSCS and TMS induced facilitation of corticospinal excitability for at least 30 min after the PAS, which is indicative of long-term potentiation (LTP)-like plasticity in the lower limb region of the primary motor cortex [81]. An important component of tSCS is its neuromodulatory effect on remote segments of the spinal cord, which must be considered during SCI rehabilitation.

## 4. Transcutaneous SCS Alters Excitability across Multiple Levels of the Spinal Cord

**Figure 3:**
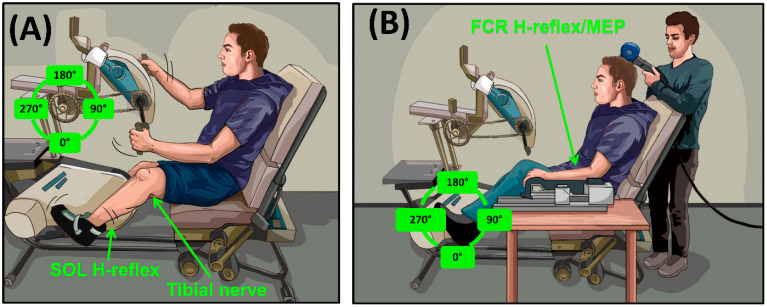
Experimental setup for investigating the effect of modulated tSCS on cervicolumbar connectivity and corticospinal facilitation: (**A**) Hoffmann (H-) reflexes were evoked during tSCS via stimulation of the tibial nerve and recorded in the soleus (SOL) muscle. The left leg was held static in an extended position, and stimulation to evoke the H-reflex was delivered with either the left arm held at 0° or during arm cycling. (**B**) H-reflexes were evoked during tSCS via stimulation of the median nerve and recorded in the flexor carpi radialis (FCR) muscle, while motor evoked potentials (MEPs) were evoked in the contralateral motor cortex and recorded in the FCR muscle, either with the legs held static, or during leg cycling. Responses were evoked during a consistent background contraction of ≈5–10% peak muscle activity at the same position, regardless of condition.

**Figure 4:**
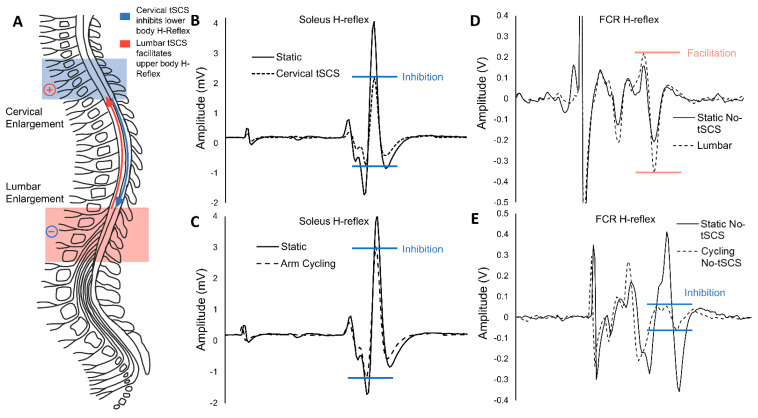
Effects of tSCS on interlimb connectivity are not similar to those of cycling in terms of reciprocal organization: (**A**) The schematic highlights common spinal segments activated by tSCS, including the cervical (blue) and lumbar (pink) enlargements. The blue arrow indicates that tonic cervical tSCS inhibits lumbar excitability, while the red arrow indicates that lumbar tSCS facilitates cervical excitability in neurologically intact individuals. (**B**) Spinal reflex excitability as assessed by the H-reflex in the soleus muscle is significantly inhibited in the presence of cervical tSCS [82]. (**C**) Spinal reflex excitability is similarly reduced in the lower limbs during arm cycling, which is a known condition for altering interlimb connectivity via presynaptic mechanisms [84,90]. (**D**) Conversely, spinal reflex excitability as assessed by the H-reflex in the FCR muscle is significantly facilitated in the presence of lumbar tSCS [70]. (**E**) Leg cycling continues to inhibit spinal reflex excitability in the upper limbs. Panels (**B**–**D**) adapted from published data in [70,82].

Evidence indicates that tSCS alters the excitability of multiple segments of the spinal cord [70,82]. These multi-segmental effects were specifically investigated in our recent work exploring how stimulation alters excitability across multiple levels of the spinal cord in neurologically intact participants, using the setup described in Figure 3. We first determined that cervical tSCS suppresses the amplitude of the soleus Hoffmann (H)-reflex by 22.9% (Figure 4B), which was similar to the 19.7% reduction produced by rhythmic arm cycling (Figure 4C), demonstrating that cervical tSCS alters lumbar excitability [59]. The suppression of H-reflexes evoked in one limb by rhythmic movements of the remote limbs demonstrates coupling between the arms and legs in humans [83,84,85]. A bidirectional linkage between the cervical and lumbar segments of the spinal cord exists during rhythmic movements in both quadrupedal mammals and humans [86,87], facilitated primarily by propriospinal connections [83,88]. Therefore, it was hypothesized that a similar reciprocal organization may also be revealed by tSCS applied to the cervical and lumbar networks, suggesting that tonic tSCS activates similar networks to those activated during rhythmic activity of the arms or legs [76,89]. In contrast to our hypothesis, lumbar tSCS significantly facilitated the amplitude of the H-reflex in the flexor carpi radialis (FCR) by 11.1% relative to no stimulation (Figure 4D), as opposed to the expected 13.6% reduction in reflex amplitude during leg cycling (Figure 4E) [69]. This indicates that separate propriospinal networks are likely responsible for the effects of tSCS and rhythmic cycling.

These results are summarized in Figure 4A, as tonic activation of spinal cord networks via tSCS alters excitability over multiple segments of the spinal cord, and is not bidirectional in its effects. The mechanisms responsible for the disinhibition of the H-reflex results between the upper and lower limbs are unknown. Facilitation of the H-reflex pathway through tSCS may be due to reduced Ia presynaptic inhibition, or to facilitation of the motor pool through activation of posterior root afferents and interneuronal projections [24]. It also remains possible that the stimulation of skin itself may be a larger contributing factor in altering the excitability of the spinal cord with tSCS than previously considered [77]. Understanding the integration of tSCS across multiple segments of the spinal cord across the range of stimulation parameters is critical in order to determine whether facilitating or inhibiting the circuitry involved is desirable based on the individual, the available technology, and the primary clinical outcome. While single-site tSCS neuromodulates remote segments of the spinal cord, multiple sites of tSCS appear to converge and facilitate the spinal and corticospinal circuitry.

## 5. Multiple Sites of tSCS Converge to Facilitate Alterations in Excitability

Further improvements to the reengagement of previously inaccessible networks may be possible using multiple stimulation sites of tSCS. Previous investigations have indicated that unmodulated tSCS delivered at the vertebral level T11 can activate the locomotor circuitry in neurologically intact study participants when their legs are placed in a gravity-neutral position [91]. Simultaneous stimulation of cervical, thoracic, and lumbar levels (i.e., C5, T11, and L1, respectively) with a carrier frequency induced coordinated stepping movements with a greater range of motion at multiple joints in five of six neurologically intact participants, compared to stimulation of T11 alone [92]. The addition of stimulation at L1 and/or at C5 to stimulation at T11 immediately resulted in enhancing the kinematics and interlimb coordination as well as the EMG patterns in proximal and distal leg muscles. Moreover, paired tSCS at the L2 and S1 segments of the spinal cord resulted in greater potentiation of the evoked response than from either site alone, indicating synergistic effects of multi-segmental pathways [93]. The interactive and synergistic effects indicate multi-segmental convergence of descending, ascending and, most likely, propriospinal influences on the neuronal circuitry during tSCS [93].

**Figure 5:**
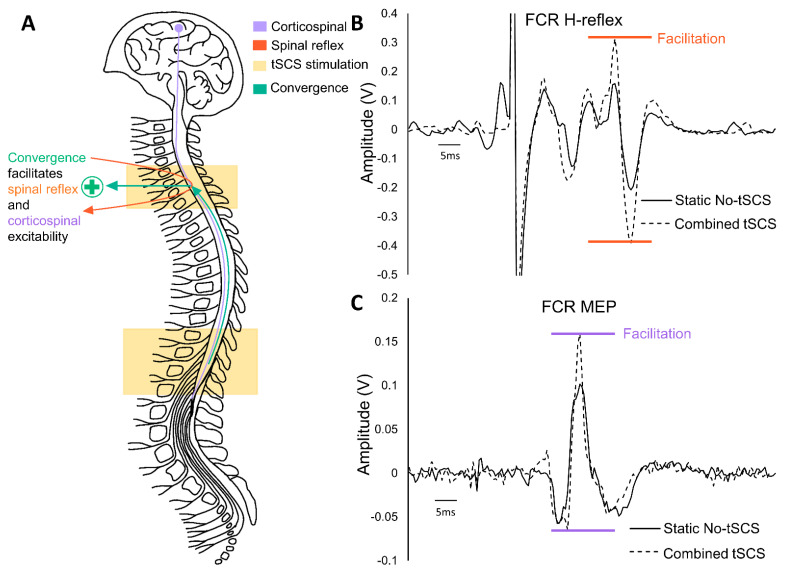
Convergence across multiple spinal segments facilitates spinal and corticospinal excitability: (**A**) The schematic highlights that simultaneous cervical and lumbar tSCS (yellow) significantly facilitates cervical spinal reflex and corticospinal excitability. (**B**) Spinal reflex excitability as assessed by the H-reflex in the flexor carpi radialis (FCR) muscle is significantly facilitated in the presence of combined cervical and lumbar tSCS. (**C**) Similarly, corticospinal excitability as assessed by MEPs in the FCR elicited from the contralateral motor cortex was also significantly facilitated by combined cervical and lumbar tSCS. Panels (**B**,**C**) adapted from published data in [69].

Interestingly, multisite (i.e., combined) modulated tSCS in both the cervical and lumbar segments of the spinal cord led to a convergence in the upper limbs (FCR muscle) that significantly increased H-reflex and MEP amplitude, by 19.6% (Figure 5B) and 19.7% (Figure 5C), respectively. Cervical tSCS alone did not increase H-reflex or MEP amplitude in the FCR, but both were significantly facilitated with the addition of lumbar tSCS. This indicates that tSCS alters excitability across multiple segments of the spinal cord, and converges to facilitate both spinal and corticospinal transmission, as demonstrated in Figure 5A. The facilitation of MEPs in the FCR by combined cervical and lumbar tSCS could be due to reinforced projection of ascending propriospinal and corticospinal axons onto cervical spinal motor neurons [94]. Therefore, the activation of proprioceptive inputs at both the cervical and lumbar spinal cord by tSCS, which synapse on cervical motor neurons, may be a major contributor to the facilitation of H-reflexes and MEPs to the FCR muscle. An important consideration with the potential use of multisite tSCS is the role that spasticity plays in the rehabilitation strategy; facilitating H-reflexes in muscles that have significant spasticity could compound the effect. Further study is required for understanding the effects of multisite tSCS in individuals living with an SCI, as well as its effects on spasticity both within a session and after training.

Interestingly, in neurologically intact study participants, modulated tSCS was unable to alter the excitability of either H-reflexes or MEPs when combined with either arm or leg cycling, regardless of whether single-site or multisite tSCS was applied [70,82]. This indicates that in neurologically intact individuals where interlimb coordination and the corticospinal tract are intact, the effects of arm or leg cycling on cervicolumbar coupling and corticospinal drive were not impacted significantly by the tSCS intensity used. Therefore, it will be a vital next step to determine the role that multisite tSCS has on interlimb connectivity after SCI. The potential impact of using multisite tSCS as a strategy to neuromodulate the spinal circuitry has significant implications in furthering our understanding of the mechanisms controlling posture and locomotion, and for regaining significant sensorimotor function even after neural injury.

## 6. Is There a Role for tSCS to Facilitate Cervicolumbar Coupling to Improve Walking?

Since single-site modulated tSCS alters excitability at remote segments of the spinal cord, and multisite modulated tSCS shows a significant convergence effect, it is possible that tSCS may influence coupling between the arms and legs after SCI. The coordination between the legs and arms is an inherent feature of locomotor neural networks [80], with coupling between the cervical (arms) and lumbar (legs) spinal networks (cervicolumbar coupling) well demonstrated in both animals and humans [95,96,97]. Oscillatory movements are governed by separate locomotor centers known as central pattern generators (CPGs), which are located in the cervical and lumbar spinal cord segments [76,89]. Similarly to quadrupedal mammals, a bidirectional linkage between the cervical and lumbar segments of the spinal cord during rhythmic movements is present in humans [86,87], facilitated primarily by propriospinal connections [83,88].

Engaging these connections with simultaneous arm and leg (A&L) cycling training improves walking capacity after both chronic incomplete SCI [98] and stroke [99,100]. Highlighting the importance of these interlimb connections, arms-only cycling has also been shown to improve overground walking function after stroke [101]. A&L cycling often capitalizes on the incompleteness of the injury to the spinal cord, even in cases where the injury is clinically classified as complete. The effect of neuromodulation is maximized when accompanied by a residual intact descending/ascending input. While the beneficial effects of rehabilitation strategies such as arm and leg cycling on cervicolumbar coupling after incomplete SCI and stroke have been outlined previously, little is known about severe cases when the injury to the spinal cord is clinically complete. Pairing tSCS with A&L cycling may allow for similar improvements in interlimb connections after complete SCI or multiple sclerosis. However, the impact of tSCS on propriospinal connectivity has yet to be investigated. Enhancing cervicolumbar connectivity by pairing A&L cycling with tSCS to improve mobility outcomes also remains a vital avenue for future research.

## 7. Trunk Stability Improvements with tSCS

While direct evidence of tSCS influencing remote segments of the spinal cord is limited, enhancing trunk stability—which is often an overlooked component—may provide indirect evidence of the influence of tSCS. Postural stability via regulation of trunk function is an integral part of locomotor control and a key element of the kinematic chain for reaching movements [102,103]. Modulation of lumbosacral networks via modulated tSCS has enabled individuals with various levels of SCI to stand without assistance from a therapist; more importantly, individuals showed improved postural control after repeated sessions of training, as demonstrated by an increased range of the center of pressure excursion during self-initiated body weight displacement [67]. As argued by the authors, biphasic pulses were perceived similarly to the sensation caused by monophasic pulses; however, biphasic stimulation could not enable unassisted standing, and was ineffective in producing motor output in the lower extremities, even at higher stimulation intensities [67]. Although critical, this observation is limited to one specific task of the lower extremity using only a modulated waveform; thus, future investigation is necessary in order to compare the effects and the underlying mechanisms of monophasic and biphasic tSCS paradigms, in an effort to uncover the best stimulation paradigm for improving functional outcomes. Moreover, modulated tSCS applied to the lumbar region increased the level of activity in the trunk muscles, adjusted the abnormal sitting posture, and extended the limits of multidirectional seated displacement, overall enhancing postural control [104]. The ability of lumbar tSCS to improve muscle activity in the trunk and postural control provides indirect evidence for tSCS inducing meaningful effects across multiple segments within the spinal cord. While further investigation is necessary in order to determine the specific pathways responsible for improved postural control, there is an additional incentive to pair tSCS with rehabilitation interventions in order to improve functions that are often overlooked in research and rehabilitation interventions.

## 8. Previously Developed Rehabilitative Approaches Are Enhanced through tSCS

Understanding the role of tSCS across multiple converging segments of the spinal cord is an important consideration when designing optimal rehabilitation interventions. The use of tSCS in conjunction with functional training appears imperative for optimizing functional recovery after SCI [17,27,32,105]. When tSCS (either modulated or unmodulated) and training are combined, functional changes emerge more rapidly and to a greater degree than with either method alone, making these strategies vital to one another’s success [27,32]. Importantly, participants with SCI previously considered to be at maximal functional capacity following walking-based therapy were able to gain significant improvements in the 6 min and 10 m walking tests after incorporating unmodulated tSCS into a paired tSCS- and walking-based therapy intervention [32]. Likewise, unmodulated tSCS as an adjunct to locomotor training was shown to improve walking outcomes in individuals with subacute motor-incomplete SCI [106]. Furthermore, pairing modulated tSCS with walking using an exoskeleton can improve lower limb coordination [105]. Positive synergistic effects of tSCS neuromodulation and previously successful rehabilitation strategies are a promising avenue for increasing what is currently possible for recovery after neurotrauma. This may be enhanced by further understanding of the unique properties of tSCS, and how it may modulate spinal circuitry differentially based on stimulation parameters and waveforms, muscles of interest, and desired functional outcomes.

## 9. Conclusions and Future Directions

Collectively, this work demonstrates that the activation of spinal cord networks with tSCS alters excitability over multiple segments of the spinal cord, with differential properties depending on the site, waveform, and parameters of tSCS. Importantly, multiple sites of tSCS converge to enhance the modulation of spinal reflex and corticospinal pathways in neurologically intact individuals. Clinical data also indicate that multi-segmental functional improvements occur after SCI. This highlights the potential for engaging cervical and lumbar spinal cord networks through tSCS to enhance the effectiveness of rehabilitation interventions. An essential next step in the evolution of tSCS research is determining the unique contributions of cutaneous mechanoreceptors, islet cells, dorsal root afferents, interneuronal projections, and large afferents in the dorsal horn, all of which likely contribute to neuromodulation with tSCS. Understanding the mechanisms of action with tSCS and potential differences in recruitment between modulated and unmodulated tSCS will provide the foundation with which to establish optimal concomitant rehabilitation therapy to improve sensorimotor function after neural injury or disease [66,107]. In general, tSCS appears to be a safe approach for modulating the excitability of neural networks of the spinal cord. Several studies have reported that the stimulation is well tolerated with minimal skin irritation or adverse changes in blood pressure, heart rate, spasticity, and/or incontinence [2,27]. However, two studies have reported side effects after tSCS including unintentional voiding during standing, skin damage and redness, fluctuation of blood pressure and heart rate, and nausea [63,105], and significant work is still required in order to ensure the safety of tSCS, including its application to locations where autonomic nerves are located. Specific caution should also be taken to ensure that tSCS is not applied to areas containing a metal implant or medical device, because the potential interactions between tSCS and such devices have not been explored [106].

## Data Availability

Processed data from reference [60] can be accessed in the Open Data Commons for Spinal Cord Injury at http://dx.doi.org/10.34945/F5B59T that was published on 9 March 2021 [108], and last accessed on 17 January 2022.
